# Therapeutic Implications for PDE2 and cGMP/cAMP Mediated Crosstalk in Cardiovascular Diseases

**DOI:** 10.3390/ijms21207462

**Published:** 2020-10-10

**Authors:** Mirna S. Sadek, Eleder Cachorro, Ali El-Armouche, Susanne Kämmerer

**Affiliations:** Department of Pharmacology and Toxicology, Carl Gustav Carus Faculty of Medicine, Technische Universität Dresden, Fetscherstraße 74, 01307 Dresden, Germany; mirna.sadek@tu-dresden.de (M.S.S.); eleder.cachorro_puente@tu-dresden.de (E.C.)

**Keywords:** PDE2, cAMP/cGMP crosstalk, natriuretic peptides, NO signaling, heart failure, arrhythmia, inflammation, cardiovascular disease

## Abstract

Phosphodiesterases (PDEs) are the principal superfamily of enzymes responsible for degrading the secondary messengers 3′,5′-cyclic nucleotides cAMP and cGMP. Their refined subcellular localization and substrate specificity contribute to finely regulate cAMP/cGMP gradients in various cellular microdomains. Redistribution of multiple signal compartmentalization components is often perceived under pathological conditions. Thereby PDEs have long been pursued as therapeutic targets in diverse disease conditions including neurological, metabolic, cancer and autoimmune disorders in addition to numerous cardiovascular diseases (CVDs). PDE2 is a unique member of the broad family of PDEs. In addition to its capability to hydrolyze both cAMP and cGMP, PDE2 is the sole isoform that may be allosterically activated by cGMP increasing its cAMP hydrolyzing activity. Within the cardiovascular system, PDE2 serves as an integral regulator for the crosstalk between cAMP/cGMP pathways and thereby may couple chronically adverse augmented cAMP signaling with cardioprotective cGMP signaling. This review provides a comprehensive overview of PDE2 regulatory functions in multiple cellular components within the cardiovascular system and also within various subcellular microdomains. Implications for PDE2- mediated crosstalk mechanisms in diverse cardiovascular pathologies are discussed highlighting the prospective use of PDE2 as a potential therapeutic target in cardiovascular disorders.

## 1. Introduction

Cardiovascular diseases (CVDs) remain a leading cause of morbidity and mortality and impose a major global health care burden. In 2017, 17.8 million deaths due to CVDs were estimated worldwide and projections anticipate a 30% increase in CVD prevalence from 2015 to 2035 [1,2,3]. Atherosclerotic and ischemic cardiac conditions contribute to CVD progression leading to the development of coronary artery disease and peripheral vascular disease. Subsequently, these conditions increase the susceptibility towards myocardial infarction (MI), cardiac arrhythmias and strokes [4]. Etiological predisposing risk factors include hypertension, hyperlipidemia, obesity, diabetes, lack of physical activity and smoking. Currently available therapeutic strategies thereby aim to prevent and limit predisposing causes. In addition to lifestyle modifications, antihypertensive, anticoagulatory and lipid lowering drugs as well as β-blockers and inhibitors of the renin-angiotensin-aldosterone system are principal pharmacotherapeutic agents commonly used in CVD patients [4,5]. Despite the effectiveness of the aforementioned medicaments, poor patient prognosis and inadequate improvement are achieved.

Present therapeutic approaches considerably neglect and overlook signalling processes occurring at the subcellular level in various cardiovascular tissues. However, increasing evidence currently points towards perturbed functional alterations in multiple cardiac subcellular microdomains [6]. Pathological redistribution of compartmentalized cellular components modulating cyclic nucleotide secondary messenger signalling are increasingly being reported to prompt pathways associated with adverse cardiac remodeling [7,8,9]. Many researchers have therefore focused to investigate compartmentalized microdomain signalling and established multiple indispensable novel technology platforms to examine complex signalling microcompartments [10,11,12]. Development of cardiac and vascular microdomain specific therapeutics may potentially offer a breakthrough in CVD therapy by limiting pathological signalling and remodeling mechanisms.

Cyclic nucleotide degrading phosphodiesterases (PDEs) are a superfamily of hydrolyzing enzymes that play an important role in the regulation of dynamic cyclic nucleotide microdomains [13]. Myocardial predominant PDE3 and PDE4 have been more comprehensively studied. Inhibitors for these PDEs, as well as for PDE5, have been recently investigated for use in multiple clinical trials [14,15]. Currently, there is increasing interest to inspect the role of PDE2 in the cardiovascular system. Several studies demonstrated the impact of PDE2 on heart rate regulation and modulation of cardiac function and remodeling mechanisms under pathological conditions [16,17,18,19]. In this review, we thereby aim to provide a comprehensive overview regarding compartmentalization mechanisms within the cardiovascular system and describe the role of PDE2 in various myocardial cellular microdomains. A synopsis of PDE2 crosstalk mechanisms identified in various cardiovascular disease settings is also discussed highlighting potential implications for the use of PDE2 as a therapeutic target.

## 2. Cyclic Nucleotide Signalling and Compartmentalization

The intracellular secondary messengers 3′,5′-cyclic adenosine monophosphate (cAMP) and 3′,5′-cyclic guanosine monophosphate (cGMP) play a central role in the cardiac and vascular signalosome, regulating multiple cellular processes under physiological as well as pathological conditions. Synthesis and distribution of both cAMP and cGMP is highly regulated by multiple complex mechanisms at a subcellular level and also diversely in different regions of the heart [6,20].

### 2.1. cAMP Signalling Pathway

Activation of G protein coupled receptors (GPCRs) by various neurotransmitters and hormones acts to modulate cAMP synthesis. Activators binding to their corresponding GPCR include: epinephrine and noradrenaline which bind to β-adrenoceptors (β-ARs) [21]; prostaglandin E1 and E2 bind to EP receptors [22,23]; glucagon binds to the glucagon receptor [24] and glucagon-like peptide-1 also activates GLP1-receptors [25]. Upon binding, Gα_s_-GPCR conformational changes trigger the activation of diverse adenylyl cyclase (AC) isoforms, converting ATP to cAMP [26,27,28]. On the other hand, soluble adenylyl cyclases (sACs) are activated by bicarbonate, enhancing the production of cAMP in the cytosol and mitochondria [29]. In general, nine AC isoforms (AC1–9) have been characterized in the human heart, with AC5 and AC6 being the most prominent in cardiomyocytes [30,31,32]. In the myocardium, sympathetic β-adrenergic signalling is the principle regulatory pathway modulating the cardiac activity upon elevating cAMP levels. cAMP-dependent activation of downstream targets increases the cardiac heart rate (chronotropic effects), contractility (ionotropic effects) and relaxation time (lusitropic effects) [33,34]. Diversely, cAMP coordinates multiple fundamental vascular processes including vascular smooth muscle cell (VSMC) proliferation [35] and vasodilatation [36] and additionally regulates endothelial barrier function [37].

The major cAMP effector target is protein kinase A (PKA). cAMP-dependent activation of PKA leads to the phosphorylation of a plethora of proteins constituting the cardiac excitation-contraction coupling (ECC) machinery. In the heart, increased cardiac contractility and heart rate are achieved upon PKA-dependent phosphorylation of L-type Ca^2+^ channels (LTCC) at the plasmalemma and ryanodine receptors (RyRs) and phospholamban (PLB) at the sarcoplasmic reticulum (SR), enhancing Ca^2+^ fluxes and cytosolic Ca^2+^ levels [33]. At the myofilaments, phosphorylation of the myosin-binding protein C (MYBPC) increases the speed of cross-bridge formation and contractile kinetics [38]. Lusitropic effects are on the other hand achieved upon PKA phosphorylation of phospholamban and troponin I, which speed up SR Ca^2+^ re-uptake and dissociation of Ca^2+^ from the myofilaments, respectively [39]. To the contrary, sustained catecholaminergic β-AR stimulation promotes maladaptive cAMP/PKA signalling [40]. PKA-dependent phosphorylation of the cAMP response element binding protein (CREB) family induces transcription of hypertrophic factors and the inducible cAMP early repressor (ICER) prompting impaired mitochondrial function and apoptosis [41,42,43]. On the other hand, elevated cAMP levels and increased PKA activity promote endothelial barrier stability and vasodilatation [44]. In VSMCs, cAMP/PKA signalling enhances SR Ca^2+^ re-uptake, increases the activity of various hyperpolarizing K^+^ channels and may decrease Ca^2+^ sensitivity of the contractile machinery to promote vasodilatation [36].

Another important cAMP downstream target is the family of exchange proteins directly activated by cAMP (EPAC). Like PKA, EPAC enhances cardiac contractility upon activation of Rap-, PLCε-, protein kinase C (PKC)- and CaMKII-mediated signalling, which subsequently increase Ca^2+^ transients and phosphorylation of RyRs and PLB. Anomalous EPAC signalling is often implicated under pathological conditions and was shown to contribute to maladaptive hypertrophic remodeling processes, fibrosis, arrhythmia and heart failure [45]. Similarly, cAMP/EPAC signalling regulates VSMC proliferation and migration and also mediates vasorelaxation upon modulation of K^+^ channel and RhoA activity [44]. In vascular endothelial cells (VECs), EPAC plays a crucial role to mediate cAMP-induced endothelial barrier strengthening. EPAC promotes tightened cadherin cell-cell junctions and prevents cadherin redistribution. Additionally, EPAC negatively regulates the pro-inflammatory JAK/STAT cascade to abrogate inflammation [44].

Furthermore, cAMP directly promotes chronotropic effects upon activating cyclic nucleotide gated ion channels (CNGCs) such as HCN channels expressed in the sinus node. cAMP-induced increase in HCN channel generated I_f_-currents enhances the sinus frequency to elicit spontaneous action potentials and hence the heart rate [46]. CNGCs are also expressed in VSMCs and VECs modulating the vascular tone [47,48]. Recently, the cAMP interacting Popeye-domain-containing proteins (POPDC) have been identified to play a critical regulatory role in cardiac pace maker cells at both the sinoatrial and atrioventricular nodes modulating cardiac conduction [49]. POPDC proteins are located in several membrane compartments interacting with TREK 1 channels, caveolin 3, dysferlin and dystrophin to maintain the cardiomyocyte structure and function [50,51].

### 2.2. cGMP Signalling Pathway (NO, Natriuretic Peptides)

In recent years, there has been increasing interest to investigate cardiac cGMP signalling potentiators due to their eminent cardioprotective role [52]. The second messenger cGMP is catalyzed from its precursor GTP by two distinct guanylate cyclase families, the soluble guanylyl cyclases (sGC) and particulate guanylyl cyclases (pGC) [53,54]. sGCs, also commonly known as NO-GCs, are activated by nitric oxide (NO) produced by NO synthases (NOS) in cardiac interstitial cells [55]. NO initiates the cGMP signalling cascade upon activation of cytosolic NO-GC1 predominantly, while NO-GC2 has been shown to play a more prominent role in other organs, e.g.—the brain [56,57]. Diversely, membrane bound pGCs are stimulated via various natriuretic polypeptide (NP) hormones [58]. Atrial (ANP) and brain (BNP) NPs are released primarily from the atria and ventricles consequently upon neurohormonal or mechanical stimulation of the myocardium (stretching or elevated blood pressure). On the other hand, the C-type NP (CNP) is mainly derived from endothelial cells of the vasculature and fibroblasts. While ANP and BNP predominantly interact with the GC-A receptor (also known as NPR-1 or NPR-A) to promote cGMP synthesis, CNP exhibits a higher affinity towards the GC-B (NPR-2 or NPR-B) receptor [58,59,60]. Both sGC and pGC generate spatially distinct cGMP signals within cardiac myocytes [61].

Upon the activation of GCs, produced cGMP acts to mediate crucial regulatory responses upon interacting with its main effector kinase PKG (protein kinase G) [62,63]. In the heart, cGMP acts to modulate cardiac contractility and enhance relaxation kinetics via PKG-dependent phosphorylation of LTCC, titin, troponin I and phospholamban [64,65]. More importantly, cGMP-mediated PKG activity was also shown to inhibit maladaptive cardiac remodeling processes. For example, cGMP/PKG-dependent phosphorylation of LTCC and TRPC6 channels subsequently suppresses the Ca^2+^-dependent calcineurin-NFAT hypertrophic pathway [66,67]. Also, PKG-mediated phosphorylation of Regulator of G protein Signalling proteins (RGS2 and RGS4) increases their activity to inhibit sustained catecholaminergic-induced Gq signalling and hypertrophy [68,69]. The cGMP/PKG signalling cascade also contributes to protect against ischemic injury and cardiomyocyte death, minimizing the infarct size after myocardial ischemia-reperfusion injury in rodents and in humans [70,71]. PKG modulation of phospholemman protects against reperfusion injury via stimulation of the Na^+^/K^+^ ATPase and thereby limits Na^+^ accumulation during cardiac ischemia [72]. Other mechanisms have also been reported, including PKG-induced opening of mitoK_ATP_ channel and upregulation of the antiapoptotic Bcl-2, thus inhibiting mitochondrial permeability transition (MPT) pore formation and cell death [73,74,75]. Thereby, the cGMP signalling cascade additionally functions as a cardiac halting or brake system ensuring appropriate cardiac function and limits pathological signal transduction.

In contrast to the heart, cGMP does not act to antagonize cAMP-mediated responses in VSMCs, however both cyclic nucleotides induce similar physiological functions. cGMP/PKG signalling is the principal modulator of the vascular tone and acts to maintain vascular homeostasis as well as cell survival [76]. This is achieved downstream upon both sGC and pGC-induced cGMP/PKG activation. PKG-mediated phosphorylation of the myosin light chain phosphatases, RhoA, RGS-2, IRAG and calcium-sensitive potassium channels (BK_Ca_) promote vasodilation [36,76].

Similar to cAMP, cGMP can partially activate CNGCs and thereby modulate beat frequency initiation in sinoatrial cells [62,77]. Moreover, cGMP is able to regulate the activity of multiple phosphodiesterase (PDE) enzymes expressed in the myocardium, providing a setting for crosstalk mechanisms between both cAMP/cGMP pathways as will be discussed in the following sections [62,78].

### 2.3. Cellular Compartmentalization Mechanisms

Despite the diverse given intracellular effects of cAMP and cGMP, distinct downstream responses are specifically provoked subsequently to various receptor stimuli. Already in 1979, contrasting physiological outcomes on cardiac contractility and intracellular signal transduction were described upon specific cardiac GPCR stimulation, although comparable cAMP levels were generated [79]. This has led to the current widely accepted model of compartmentalized cyclic nucleotide signalling. The model presumes that distinct responses are achieved upon the activation of specific signal transduction components coordinating cAMP/cGMP gradients including receptors, modulators and targets. Such components may be assembled in macromolecular complexes that are spatially confined within discrete subcellular domains, thus allowing for selective target activation [80]. To the contrary, loss of subcellular microdomain signalling mechanisms, upon redistribution of compartment restricted components, is perceived in multiple pathophysiological conditions [7,81]. Multiple subcellular components and mechanisms function to define and preserve localized cAMP and cGMP pools, including (i) localization of plasma membrane receptors, (ii) confined distribution of effector targets and (iii) localization of elements restricting secondary messenger diffusion.

(i) Characteristic cardiomyocyte transverse tubules (T-tubules), caveolae and non-caveolae membrane microdomains contribute to ensure compartmentalized signal initiation [82]. Importantly, cholesterol and sphingolipid rich areas in the membrane, called lipid rafts, form gel-like, liquid-ordered domains that hinder membrane fluidity and prevent localized GPCRs to freely diffuse throughout the lipid bilayer [83]. Modern tools combining the use of scanning ion-conductance microscopy (SICM) and Förster resonance energy transfer (FRET) techniques aided to identify domain specific cAMP fluctuations upon generating a detailed topographical image of the cell surface. β_1_-AR specific cAMP responses were thereby detected at both T-tubule and non-T-tubule microdomains, whereas cAMP synthesis upon β_2_-AR specific activation was confined to the T-tubules only [8]. In a similar manner, cGMP responses after specific β_3_-AR stimulation were also observed to be restricted to the T-tubules [84]. Besides their influence on membrane fluidity, caveolae/lipid rafts localized within T-tubule and non-T-tubule regions also function to define AC isoform localization. In various cell types, AC1, AC3, AC5, AC6 and AC8 were reported to associate with lipid raft domains, while AC2, AC4, AC7 and AC9 were identified within non-raft microdomains [85]. Other studies also demonstrated that the β_2_-AR macromolecular complex including Gs, Gi, AC5, AC6 and PKA is specifically localized within caveolar membrane fragments. Contrastingly, β_1_-ARs are ubiquitously distributed in both caveolar and non-caveolar compartments in adult rat ventricular myocytes [86,87,88,89]. Furthermore, the β_3_-AR/eNOS/sGC macromachinery was also demonstrated to be localized within caveolae-enriched membrane fractions. On the other hand, heart failure contributes to β_3_-AR redistribution and altered co-localization of sGC and caveolin-3, disrupting compartmentalized cGMP synthesis [84]. Moreover, caveolae play a fundamental role to mediate appropriate signalling responses in VSMCs and the endothelium. In caveolin-1 deficient mice, impaired endothelium-dependent relaxation and reduced myogenic tone were observed [90,91]. Also, Sampson et al., demonstrated that K_ATP_ channels and AC co-localization in rat aortic smooth muscle caveolae is crucial for appropriate K_ATP_ channel modulation [92].
(ii) Compartmentalized signalling also relies on the spatial organization of intracellular molecules coupling PKA activity to downstream effector targets as well as signal feedback regulatory elements. A-kinase anchoring proteins (AKAPs) are a superfamily of organizing scaffold proteins which are able to bind PKA and other signalling enzymes, directing their localization to specific cellular compartments [32,93,94]. 17 AKAPs have been detected and identified in cardiac tissues coordinating a plethora of signalling components besides PKA including PKC, PDEs, ACs, phosphatases and GTPases. Accordingly, their function has been highlighted to partake in various homeostatic as well as cardioprotective processes including calcium cycling, ECC coupling, heart rhythm and action potential regulation. Additionally, AKAPs coordinate signalling cascades involved in cardiac remodeling responses under pathophysiological settings [94,95,96,97]. Several AKAP-specific interactions with multiple AC and PDE isoforms have been identified, supporting precise compartment boundary construction [98,99,100]. Among others, AKAP79/150 was identified to associate with AC5 and AC6 [98] and mAKAP with AC5 [101]. Moreover, mAKAP was also demonstrated to associate with PDE4D3 [102,103]. Terrenoire et al., could also show that AKAP9 selectively formulates a complex between PDE4D3 and cardiac I_Ks_ channels, but not with PDE4D5 [104]. AKAP75 expression has also been demonstrated in VSMC promoting cAMP/PKA signalling [105]. AKAP-dependent PKA interactions with several ion channels (LTCC and K_ATP_) have also been described in vascular tissue [106,107]. Such highly selective interactions mediated via AKAP scaffolds certainly fine tune the elicited intracellular responses. However, further investigations are still required to fully identify specific complex interactions and elucidate their role in cAMP/cGMP signal propagation.
(iii) Localized cyclic nucleotide production alone remains insufficient to account for compartmentalized signalling responses if their diffusion throughout the cytosol is not restricted. Physical cytosolic barriers, cAMP buffering and export mechanisms via cardiac MRP4 efflux protein have been reported to limit cAMP diffusion in the cytosol [108,109]. More importantly, confined cyclic nucleotide distribution via PDE-mediated hydrolysis has been demonstrated in multiple studies [110,111]. 11 PDE subfamilies with over 100 different isoforms and splice variants have been identified [112]. Of the PDE superfamily, PDE1, 2, 3, 4, 5, 8 and 9 are fundamental constituents of the cardiac signalosome coordinating cardiac function under both physiological and pathophysiological conditions [113]. On the other hand PDE1, 2, 3, 4, 5 and 7 constitute the major PDE activity in the vasculature [114,115]. Only PDE2, PDE3 and to a lesser extent PDE1 have been shown to mediate a cGMP/cAMP crosstalk upon modulation of the PDE activities via cGMP. cGMP competitively inhibits the cAMP hydrolytic activity of both PDE1 and PDE3. Exceptionally, PDE2 is the only member of the PDE family that is activated upon allosteric cGMP binding, increasing its cAMP hydrolytic activity [110]. In this review, we will particularly focus to discuss PDE2 functional role in the cardiovascular system.

### 2.4. PDE2 Molecular Aspects and cGMP/cAMP-Mediated Crosstalk

PDE2 was first characterized in 1981 as the cGMP-stimulated PDE with dual-substrate specificity for both cGMP and cAMP [116]. PDE2 exhibits similar hydrolyzing maximal velocities and K_m_ for both cGMP (10µM) and cAMP (30 µM). However in the presence of cGMP, PDE2 hydrolytic activity towards cAMP is uniquely enhanced ~5–6-folds [116]. Native human PDE2 is a protein of 941 amino acids, which functions as a homodimer and is organized in four domains: *N* terminus (1–214), GAF-A (215–372), GAF-B (393–541) and the catalytic domain (579–941) [117]. So far, two PDE2 genes, PDE2A and PDE2B, have been identified; however PDE2B exists in non-mammalian species and has been characterized in *Trypanosoma brucei* parasites [118]. Upon transcription, three different splice variants PDE2A1, PDE2A2 and PDE2A3 are constructed [119]. While the three splice forms share similar domain structures, differences arise within the *N*-terminal domain designating their intracellular localization [119,120]. In cardiomyocytes, PDE2A1 was reported to localize in the cytoplasm while PDE2A2 and PDE2A3 are exhibited in particulate and membrane fractions [121]. PDE2A2 localization seems to be confined to the mitochondrial compartment, influencing mitochondrial respiration processes [122]. However, PDE2A has been generally detected in nuclear envelope, Golgi body, plasma membrane and sarcoplasmic reticulum fractions [123,124,125].

To function as a homodimer, PDE2 monomer dimerization is accomplished via the GAF-A binding locus [126]. The GAF-B domain, on the other hand, modulates PDE2 activity upon cGMP binding [126,127]. At sub-saturating cAMP concentrations, cGMP interactions at the GAF-B domain prompt conformational changes at the PDE2 catalytic site leading to a considerable increase in cAMP hydrolysis [126]. Similarly, cAMP can also bind to GAF-B mediating conformational changes, however with a much lower affinity when compared to cGMP (~21-fold lower). The ability of PDE2 to discriminate between cGMP and cAMP might be in part due to interactions with two amino acids (Phe-438, Asp-439) which enhance cGMP binding but do not favor cAMP interactions [128].

Similar to other PDEs, the alpha-helical architecture of the catalytic domain includes two metal ions, Mg^2+^ and Zn^2+^, embedded within the active site in coordination with histidines, aspartic acids, and water molecules [129,130]. Diversely, PDE2 uniquely holds two major structural differences within its catalytic site when compared to other PDEs. First, the H-loop has a conformation that results in the occlusion of the substrate-binding site. Secondly, the M-loop is folded away from the substrate-binding site and participates in dimer contacts. In other PDEs, the M-loop is normally folded in towards the binding site. These characteristic conformational properties of PDE2 allows the substrate-binding areas of the monomers to face each other, closing off the access to the substrate-binding site. In this situation, the domain appears to be in a “closed” configuration. However, after cGMP binding to the GAF-B domain, the H-loop swings away and the catalytic domain adopts an “open” configuration, allowing the substrate to bind [117]. Moreover, cAMP and cGMP interact with three hydrophobic sub-pockets within the active site: the pocket containing the glutamine-switch and hydrophobic clamp (Q-pocket), the metal binding pocket (M-pocket), and the solvent-filled side pocket (S-pocket) [129,130]. In general, various studies have concomitantly shown that PDE2 can hydrolyze both cAMP and cGMP with similar affinities and kinetics in various myocardial-derived cellular fractions and tissues [131]. However, at higher concentrations each cyclic nucleotide may act as a competitive inhibitor for the other’s hydrolysis. At sub-saturating concentrations, cGMP enhances cAMP hydrolysis; however, the reverse may also hold true. Nevertheless, the assumption of cAMP-stimulated cGMP hydrolysis is not very likely to occur in vivo due to the lower cAMP affinity to the GAF-B domain [128,132].

In addition to cyclic nucleotides, PDE2 has also been reputed to associate with scaffold proteins via high-throughput screening studies [133]. PDE2 was shown to interact with XAP2, a crucial component of the aryl hydrocarbon receptor (AhR) complex playing an important role in cardiomyocyte differentiation and hypertrophy [134,135]. XAP2 targets PDE2 to the AhR complex and thereby inhibits the nuclear translocation of AhR. The carboxyl-terminal part of XAP2 docks to the GAF-B domain of PDE2; nevertheless, allosteric cGMP interactions at the GAF-B domain are not affected [134].

**Figure 1:**
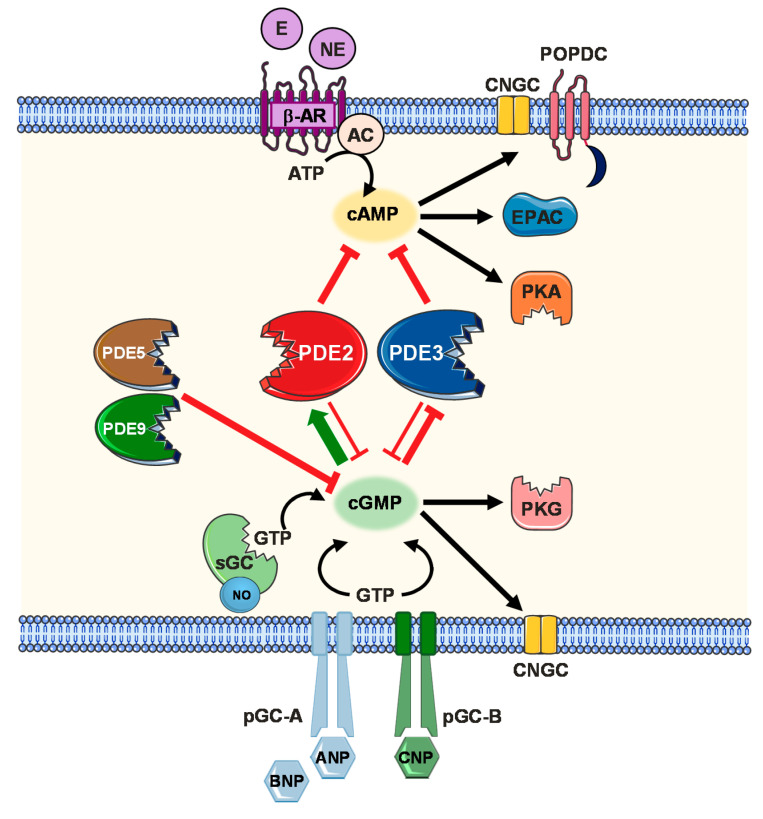
Simplified scheme of phosphodiesterase (PDE)-mediated cGMP/cAMP crosstalk. cAMP generated by adenylate cyclase (AC) upon stimulation of β-adrenergic receptors leads to the activation of protein kinase A (PKA) and the family of exchange proteins directly activated by cAMP (EPAC). cGMP generated either by NO-sensitive soluble guanylyl cyclase (sGC) or natriuretic peptide (NP)-sensitive GC-receptors leads to the stimulation of cGMP-dependent protein kinase G (PKG). In diverse subcellular compartments, cGMP stimulates phosphodiesterase 2 (PDE2) but competitively inhibits PDE3-mediated cAMP hydrolysis. PDE5 and PDE9 degrade cGMP in cardiomyocytes. Figure was produced using Servier Medical Art (Available online: http://smart.servier.com).

The crosstalk between both cAMP and cGMP pathways is complex and differentially regulated by multiple factors in diverse cardiovascular cell types and within different subcellular microdomains. Crosstalk mechanisms are influenced by the relative abundancy of local cyclic nucleotide levels, in addition to the phosphodiesterase isoforms expressed within a specific microcompartment. At low cGMP concentrations, PDE3 is competitively inhibited limiting its cAMP hydrolytic activity. On the other hand, intermediate levels of cGMP allosterically activate PDE2 mediating a negative cGMP/cAMP crosstalk. At higher cGMP concentrations, PDE1 is potentially inhibited [110,136]. Other phosphodiesterases expressed within the myocardium also effectively contribute to regulate cyclic nucleotide levels and have already been investigated as potential therapeutic targets in cardiac disease at a clinical level, e.g., PDE5 [14,137,138]. Hereby, Figure 1 illustrates a simplified overview of complex cAMP and cGMP crosstalk mechanisms mediated via PDE2 and influenced by other PDE family members expressed in the cardiovascular system. Nevertheless, multiple studies specifically provided strong evidence for PDE2-mediated negative cGMP/cAMP crosstalk mechanisms in various cardiac cells. For example, NO-stimulated cGMP generation was shown to activate PDE2, enhancing cAMP hydrolysis in neonatal cardiomyocytes as well as in guinea pig pacemaker cells, rat cardiac fibroblasts and human atrial cardiomyocytes [61,139,140,141]. Additionally, NP/cGMP-induced stimulation of PDE2 enhancing cAMP degradation was reported in rat myocardium [142]. Physiological and pathophysiological consequences of this crosstalk will be further reviewed below.

## 3. PDE2 Functions in the Cardiovascular System

Within the cardiovascular system, PDE2 is predominantly expressed in endothelial cells and in cardiac fibroblasts but is modestly expressed in cardiomyocytes under physiological conditions [133,143,144]. Prominent PDE2 expression is notable in various neuronal cell types within the central nervous system and thereby PDE2 activity in sympathetic neurons also modulates the cardiac function [145,146,147,148]. Furthermore, PDE2 activity was also demonstrated in circulating immune cells, e.g., monocytes, macrophages and lymphocytes [149,150,151,152]. In human and rodent monocytes, the macrophage colony-stimulating factor (M-CSF) was shown to induce PDE2 expression in monocytes differentiating in peritoneal macrophages [149,150]. Additionally, the cytokine tumor necrosis factor-α (TNF-α) was also reported to induce the expression of PDE2 and contribute to increase endothelial permeability [153]. Thereby, in the following sections we will focus to discuss the function of PDE2 within the myocardial tissue under both physiological and pathophysiological conditions.

### 3.1. Cardiomyocytes

**Figure 2:**
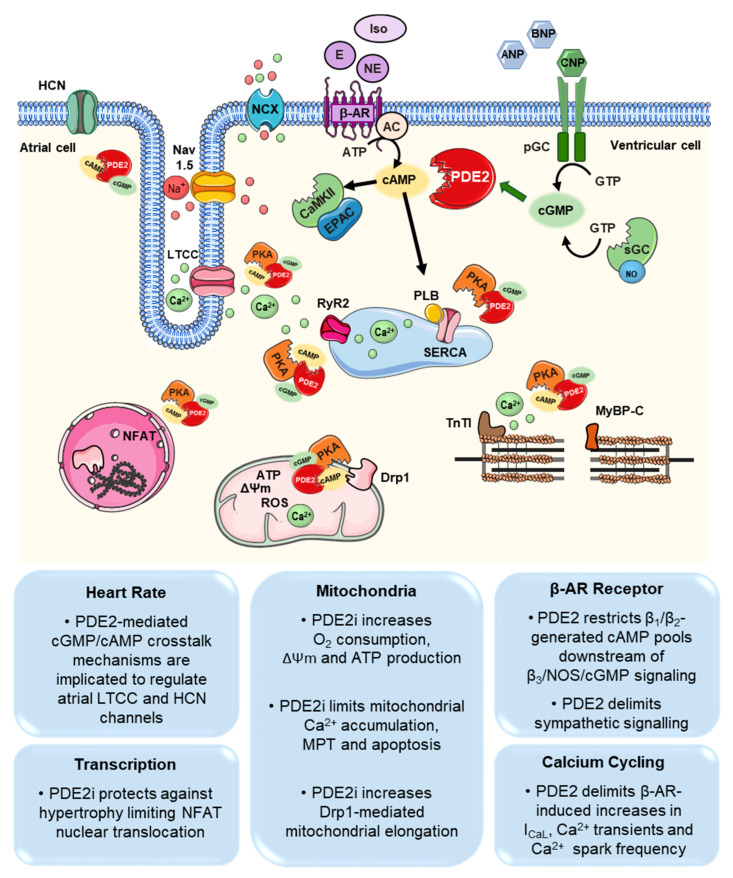
PDE2-mediated cGMP/cAMP crosstalk mechanisms in cardiomyocytes. PDE2 regulates cAMP levels downstream of β-AR signalling at a compartmentalized level, modulating PKA- and potentially EPAC/CaMKII-mediated activation of numerous targets. First, PDE2 regulatory activities are detected at the plasmalemma, modulating I_Ca,L_ and possibly HCN I_f_ currents. Additionally, PDE2 regulates components of the ECC machinery at the SR (RyR2 and PLB) and sarcomeres (TnT1 and MyBP-C). PDE2 transcriptional regulatory effects are also reported in the nuclear compartment in addition to its role to regulate mitochondrial function and dynamics. PDE2 is also activated by cGMP synthesized downstream of both sGC and pGC, to promote a negative cGMP/cAMP crosstalk mechanisms in the aforementioned compartments. (PDE2 inhibition (PDE2i)) Figure was produced using Servier Medical Art (Available online: http://smart.servier.com).

Despite the limited expression of PDE2 in cardiomyocytes and its modest contribution to the total cAMP hydrolytic activity (~3%) under physiological conditions, PDE2 remains a well-recognized and important modulator of cardiac function [131,133,154]. PDE2 has been shown to finely coordinate critical cAMP pools governing β-AR signalling, cardiomyocyte contractility, Ca^2+^ homeostasis and mitochondrial function (Figure 2) [122,154,155].

PDE2 seems to closely couple with the β-AR family in cardiomyocytes. Mongillo et al., could show that PDE2 finely coordinates β_1_/β_2_-AR-generated cAMP pools downstream of the β_3_-AR/NO pathway. While PDE2 inhibition leads to a minor increase in intracellular cAMP levels under basal conditions, a marked increase in norepinephrine-induced [cAMP]_i_ responses was observed following PDE2 inhibition (~7-folds). Hereby, the abolished cAMP restriction indicates that β-AR–generated cAMP pools are strictly defined by PDE2. A key mechanism partly contributing to increased PDE2 activity within this compartment was attributed to norepinephrine-mediated activation of β_3_-AR. This subsequently potentiates the eNOS/NO/cGMP signalling cascade and activation of PDE2 [124]. In heart failure, Schobesberger et al., could demonstrate that PDE2-mediated cGMP/cAMP crosstalk mechanisms downstream of β_3_-AR signaling pathways were impaired due to altered β_3_-AR and sGC distribution [84].

The regulatory role of PDE2 in shaping β-AR responses is also associated with refined phosphorylation of downstream kinase targets like LTCCs, troponin I and MYBPC [155,156]. Earlier investigations highlighted a role for PDE2 in L-type Ca^2+^ current (I_Ca,L_) modulation, particularly in the human myocardium. Rivet et al., could display an increase in I_Ca,L_ upon PDE2 selective inhibition with EHNA in human atrial myocytes [157]. Similarly, multiple groups also indicated reduced I_Ca,L_ upon cGMP-induced activation of PDE2. Reduced I_Ca,L_ in this setting was attributed to lower LTCC phosphorylation at PKA specific sites due to the diminished cAMP levels in this compartment [141,156,158]. Consequences of attenuated I_Ca,L_ upon PDE2-mediated cAMP degradation are associated with the regulation of cardiac heart rate and cardiomyocyte contractility. In pacemaker cells, NO-induced heart rate modulation was shown to be partly dependent on cGMP/PDE2 activation and finally I_Ca,L_ attenuation [110,159]. Furthermore, Mongillo et al., demonstrated that PDE2 potently modulates cardiomyocyte contractility following β-AR stimulation in rat ventricular myocytes [124]. The increased cGMP synthesis following sodium nitroprusside (SNP) treatment provoked PDE2 activation and consequently abrogated norepinephrine-induced increase in cAMP amplitudes by 50%. Thereby, PDE2 inhibition amplified norepinephrine- and isoprenaline-induced increase in Ca^2+^ transients and fractional shortening [124]. Likewise, Mika et al., showed increased Ca^2+^ transients, sarcomere shortening and enhanced MYBPC3 phosphorylation after specific PDE2 inhibition with BAY60-7550 [155]. Correspondingly, mice with cardiac-specific PDE2 overexpression exhibited lower heart rates and displayed blunted isoprenaline-induced increases in cAMP levels as well as in I_Ca,L_, Ca^2+^ transients and sarcomere shortening [17]. Redistribution of PDE2, as well as PDE3, within β-AR microdomains has been reported in heart failure animal models, influencing cardiac responses and contractility [7,160]. Relocalization of PDE2 from β_1_-AR–associated noncaveolar into β_2_-AR–containing caveolar fractions was demonstrated in cardiac hypertrophy after transaortic constriction [7]. Additionally, the PDE3-induced hydrolysis of cAMP downstream of β_2_-ARs within the phospholemman/sodium-potassium ATPase microdomain was diminished after MI-induced chronic heart failure in rats. However, a significant increase in PDE2-mediated effects was thereafter recognized in this compartment confirming PDE2 and PDE3 relocalization in heart failure [160].

Apart from β-AR signalling and ECC regulatory functions, PDE2 is also associated with critical signalling pathways modulating cardiomyocytes’ energetic capacities and apoptosis. Liu et al., highlighted that in the presence of cGMP, PDE2 holds the largest mitochondrial cAMP-degrading activity when compared to PDE3 and PDE4 in adult rat ventricular myocytes. They also demonstrated the PDE2 specifically localizes within subsarcolemmal mitochondria associated with the mitochondrial inner membrane [161]. Upon PDE2 inhibition with BAY60-7550, elevated mitochondrial cAMP levels were recognized, implicating an increase in oxygen consumption, mitochondrial membrane potential (ΔΨm) and ATP production [161,162,163]. Additionally, cAMP is rate-limiting for matrix Ca^2+^ entry via the exchange factor EPAC1 and the mitochondrial Ca^2+^ uniporter and thus abrogates mitochondrial permeability transition (MPT). Collectively, the latter effects contribute to protect against cardiomyocyte cell death and apoptosis [163]. Accordingly, mice with cardiomyocyte specific PDE2 overexpression exhibited faster ∆Ψm loss and mitochondrial swelling [161]. PDE2 was further shown to mediate intracellular effects of estrogens. In a rat cardiomyoblast cell line, 17β-estradiol reduced mitochondrial cAMP levels via cGMP-mediated stimulation of PDE2, finally decreasing cytochrome oxidase activity and mitochondrial membrane potential [164].

On the other hand, Monteresi et al., showed that mitochondrial PDE2A2 regulates cAMP pools localized at the outer mitochondrial membrane. This specific cAMP compartment was found to modulate the activity of the dynamin related protein 1 (DRP1) fission protein. PDE2 inhibition, and thereby elevated cAMP levels, enhanced PKA-dependent phosphorylation of DRP1. Elongated mitochondria were therefore notable due to diminished DRP1 fission activity. With such an elongated morphology, mitochondria exhibited increased ΔΨm, protecting the cardiomyocytes against apoptotic cell death [122].

### 3.2. Fibroblasts

**Figure 3:**
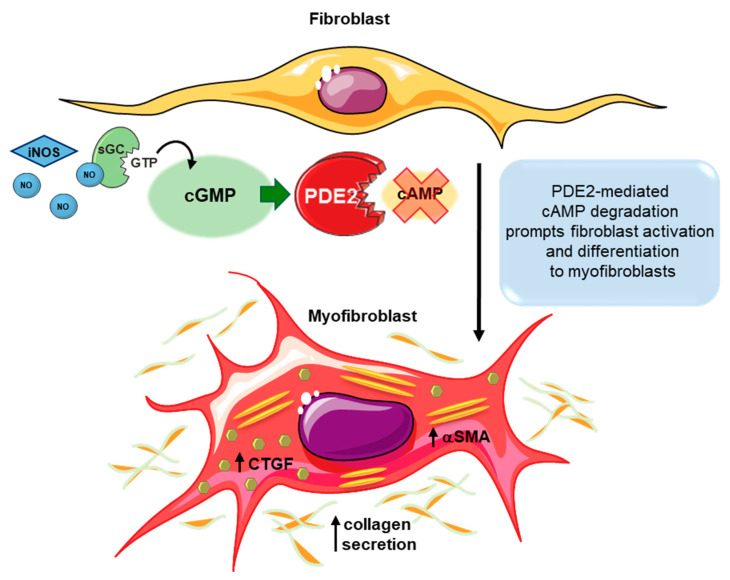
Schematic illustration of the PDE2-mediated cGMP/cAMP crosstalk in cardiac fibroblasts. PDE2 overexpression or enhanced activity subsequent to iNOS/NO/cGMP stimulation accelerates cAMP hydrolysis in cardiac fibroblasts. Diminished cAMP levels prompt fibroblast activation and differentiation into myofibroblasts promoting α-smooth muscle actin (αSMA), connective tissue growth factor (CTGF) and collagen disposition, increasing muscle stiffness. Figure was produced using Servier Medical Art (Available online: http://smart.servier.com).

PDE2 is more abundantly expressed in cardiac fibroblasts when compared to cardiomyocytes. Thereby, studying PDE2 functions in fibroblasts is of importance since fibroblasts constitute the major nonmyocyte component of the cardiac tissue comprising 65% of the heart cell number [165]. Early studies clearly highlighted PDE2-mediated cGMP/cAMP crosstalk mechanisms in adult rat fibroblasts, particularly in an inflammatory milieu. Gustafsson and Brunton first demonstrated that IL-1β induces iNOS expression in cardiac fibroblasts. Simultaneous IL-1β treatment and β-AR stimulation was found to stabilize iNOS mRNA, increasing the IL-1β-induced NO-production in the fibroblasts [166]. Later on, the authors provided evidence for an induction of a PDE2-dependent cGMP/cAMP crosstalk under these conditions. IL-1β-induced iNOS expression enhances NO/cGMP production and in turn activates PDE2, attenuating cAMP accumulation after isoprenaline or forskolin treatment [140]. However, reduced cAMP levels may mediate alternative consequences in distinct cell types of the myocardium that may not always be favorable. In fibroblasts, diminished cAMP signalling promotes fibrotic effects, cell proliferation and collagen production [167]. In line with these publications, PDE2 overexpression in cardiac fibroblasts accelerated cAMP degradation and subsequently supported fibroblast activation and transformation into myofibroblasts. Increased αSMA and CTGF profibrotic factors were observed upon PDE2 overexpression (Figure 3). These events finally resulted in higher stiffness of fibroblast-derived engineered connective tissues [144]. Therefore, PDE2 activity in distinct cardiac cells displays controversial effects within the myocardium.

### 3.3. Sympathetic Neurons

As previously introduced, PDE2 plays a crucial role in cardiac sympathetic neurons [145]. In dissociated sympathetic neurons from stellate ganglia, adenoviral-nNOS overexpression augmented cGMP levels; however, cAMP levels were consequently reduced. Diminished cellular cAMP levels subsequently inhibited Ca^2+^-influx via voltage-gated Ca^2+^ channels and thereby decreased exocytotic norepinephrine release. Adenoviral-nNOS-induced reduction in cAMP levels was reversed when cells were treated with the PDE2 inhibitor BAY60-7550, suggesting that PDE2 is the main cAMP-hydrolyzing enzyme in these cells [145]. PDE2 activity was also reported to be increased in stellate ganglion derived from patients with a sympathetic overdrive and ventricular arrhythmias. This also implicates that selective targeting of neuronal PDE2 may offer a novel potential therapeutic strategy in sympathetic hyperactivity disease settings [147].

### 3.4. Vasculature and Circulating Blood Cells

As summarized previously, PDE2 activity was detected in circulating immune cells, e.g., monocytes, macrophages and lymphocytes [149,150,151,152]. Additionally, along with PDE3 and PDE5, PDE2 is also expressed in platelets playing an important role to regulate platelet activation. RNA-sequencing analysis revealed that PDE2A2 is the predominantly expressed variant of PDE2 in human platelets [168,169]. Both cAMP and cGMP inhibit multiple signalling cascades involved in platelet activation, including degranulation, fibrinogen receptor activation, cytoskeletal rearrangement and expression of proinflammatory mediators. Thereby PDE inhibitors have been considered for antiplatelet therapy [170]. Antiplatelet effects of PDE3 and PDE5 inhibitors have been proven clinically and currently approved for intermittent claudication (cilostazol, PDE3 inhibitor) and coronary vasodilatation with antiplatelet activity (Dipyridamole, PDE3/5 inhibitor) [169,170]. PDE2 inhibitors have not been tested for clinical use; however, preclinical studies highlighted attenuated platelet activity upon PDE2 inhibition. PDE2 inhibition with EHNA was demonstrated to potentiate the inhibitory effects of nitroprusside on thrombin-induced platelet aggregation [171]. The effect of other PDE2 inhibitors, such as BAY60-7550 and PDP, on platelet aggregation remains to be investigated. Further studies are needed to prove if PDE2 might be a potential target for antiplatelet therapy in thrombotic and ischemic conditions.

**Figure 4:**
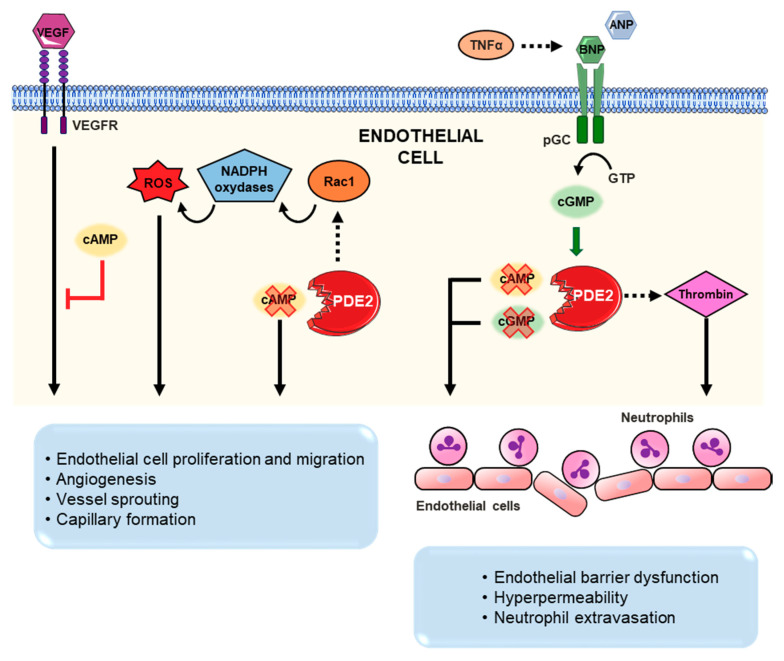
Schematic illustration of the PDE2-mediated cGMP/cAMP crosstalk in endothelial cells. PDE2 stimulates endothelial cell proliferation and angiogenesis by hydrolyzing cAMP (which reduces cell proliferation) and stimulating ROS production through Rac1 and NADPH oxidases, inducing cell proliferation and angiogenesis together with VEGF. TNF-α promotes BNP, increasing cGMP level; consequently, cGMP activates PDE2 cAMP and cGMP hydrolyzing activity and PDE2 also stimulates thrombin. This results in enhanced neutrophil infiltration, increasing inflammation. Figure was produced using Servier Medical Art (Available online: http://smart.servier.com).

Although PDE2 is not primarily expressed in vascular smooth muscle cells, PDE2 is rather highly expressed in endothelial cells [76,143,172]. A comprehensive discussion of PDE2 effects in endothelial cells and its impact on angiogenic and inflammatory responses under ischemic and septic cardiac conditions is elaborated in Section 4.3, Section 4.4 and Section 4.5 and illustrated in Figure 4.

## 4. Role of PDE2 in Cardiovascular Disease

### 4.1. Arrhythmia (Atria, Ventricular, Sinus Node)

Onset of lethal cardiac arrhythmias leading to sudden cardiac death is the most frequent cause of mortality in heart failure patients. Unfortunately, currently available antiarrhythmic pharmacotherapies are of low therapeutic indices and often intrinsically dispose proarrhythmogenic effects upon brief use [173]. The clinical use of some PDE inhibitors in heart failure, such as milrinone (PDE3 inhibitor), is limited as they have been associated with increased arrhythmogenic susceptibility [174]. Contrastingly, PDE2 seems to play a unique role in heart rate regulation and appears to provide protection against arrhythmia onset [17].

Recently, Vettel et al., could show that pharmacological PDE2 inhibition in vivo lead to an exclusive elevation in heart rates of beagle dogs and amplified β-AR-induced chronotropic responses in mice. Accordingly, transgenic animals with cardiomyocyte specific PDE2A3 overexpression (TG) exhibited lower basal heart rates. More importantly, increased PDE2A3 abundancy protected the animals against arrhythmogenesis upon cardiac stress. TG animals were resistant to triggered ventricular arrhythmias induced by catecholamine injections. Moreover, PDE2A3 overexpression remarkably protected TG mice against arrhythmia-induced early death after MI. These intriguing results were attributed to reduced RyR2 phosphorylation at the pro-arrhythmic CaMKII specific site (RyR2 Ser2814) as well as reduced I_Ca,L_ and Ca^2+^ transients achieved after β-AR stimulation. The authors could thereby confirm that PDE2A3 overexpression protected against catecholamine-induced increase in RyR2 leak; and thereby lower Ca^2+^ wave and spark frequencies were observed in cardiomyocytes isolated from TG animals when compared to their wild type littermates [17].

Given the limitations of transgenic overexpressing mouse models, particularly in the case of PDEs, where isoform specific compartmentalized expression may be perturbed, supporting investigations in PDE2-KO animals are still required. Unfortunately, global PDE2 deletion resulted in premature embryonic death (before E16). Nevertheless, documented findings in PDE2-KO embryos highlighted a critical role for PDE2 in heart rhythm regulation and cardiac development. Isolated cardiomyocytes from the fetal PDE2-KO hearts indicated higher beating frequencies; but beating was arrested more frequently when compared to control animals suggesting an increased susceptibility towards arrhythmias. Moreover, PDE2 deficiency led to embryonic nuchal edema and thereby enlarged hearts were recognized. Also, diminished atrial chamber trabeculae, impaired interventricular septum and myocardial wall defects were detected in KO embryos [175].

With the eminent impact of PDE2 on heart rate regulation and arrhythmia susceptibility, a regulatory role for PDE2 at the sinus node is suspected. Hua et al., could show that PDE2, and also PDE4, play a role in modulating action potential firing frequency in isolated cardiomyocytes from mice. Both PDEs regulate the I_Ca,L_ more prominently in murine cardiomyocytes from sinus node and atrium compared to ventricular myocytes [176]. Another study implicated a possible role for PDE2 in the regulation of the sinus hyperpolarization-activated current (I_f_). The investigators demonstrated that SNP/NO/cGMP-induced a transient increase in heart rate upon simultaneous β-AR stimulation due to potential cGMP-dependent stimulation of I_f_. Additional PDE2 inhibition amplified SNP- mediated heart rate responses, implicating that cGMP-induced activation of PDE2 limits SNP-induced heart rate effects [139]. On the other hand, PDE2A3-TG did not highlight altered activity of the cyclic nucleotide gated HCN channels. A similar decrease in heart rate was observed upon HCN blockade with ivabradine in both PDE2A3-TG and WT littermates, rather indicating an impact of PDE2 on sinus Ca^2+^ clock [17]. Further studies are needed to evaluate the direct influence of PDE2 inhibition on HCN activity expanding our understanding regarding the role of PDE2 on heart rate regulation. Nevertheless, in the murine sinoatrial node, a crosstalk between the cGMP-degrading PDE5 and cGMP-stimulated PDE2 was demonstrated to modulate β-AR-induced chronotropic responses. Isidori et al., described that specific cAMP pools generated downstream of β_2_-ARs are finely regulated by PDE2 and indirectly by PDE5. Inhibition of PDE5 promoted cGMP-dependent activation of PDE2. Thereafter, β-AR-generated cAMP pools were attenuated mediating negative chronotropic effects with concomitant modulation of calcium transients [177]. Intact PDE2 activity is therefore crucial to maintain appropriate heart rate responses to various neurohormonal stimuli.

### 4.2. Hypertrophy, Heart Failure

The role of PDE2 in heart failure and cardiac remodeling remains obscure. Contradictory findings in diverse experimental settings reflect the complex integrity of PDE2 and its involvement in regulation of multiple signalling cascades that may seem to counteract each other. Mehel et al., were the first to point out that PDE2 is upregulated in the human failing myocardium [16]. Patients suffering from end-stage dilated cardiomyopathy or ischemic cardiomyopathies exhibited ~2-fold increase in PDE2 protein levels when compared to the non-failing control group. However, PDE2 expression was not altered in hypertrophied myocardium from patients with preserved cardiac function (ejection fraction > 50%) who underwent aortic valve replacement due to aortic stenosis [16]. In diverse experimental animal models, PDE2 upregulation was shown to be closely associated with cardiac disease progression, cardiac arrhythmia onset and heart failure. PDE2 expression was elevated in a canine model of rapid pacing-induced heart failure as well as in rodent models subjected to chronic minipump catecholamine infusions or pressure overload via transverse aortic constriction [16,178]. These findings implicated that PDE2 contributes to the myocardial β-AR desensitization process and protects the damaged heart from excessive sympathetic stress. Blunted β-AR responses were confirmed upon adenoviral PDE2A2 overexpression in adult rat ventricular myocytes, which resulted in reduced cAMP levels, I_Ca,L_, Ca^2+^ transients and abrogated inotropic effects following acute β-AR stimulation without affecting basal contractility. Notably, adenoviral PDE2A2 overexpression also protected the cardiomyocytes against norepinephrine-induced hypertrophic growth [16].

To the contrary, opposing results were presented by Zoccarato et al., highlighting hypertrophic responses upon adenoviral overexpression of the PDE2A2 in neonatal rat ventricular cardiomyocytes. The group displayed that rather PDE2 inhibition counteracted the hypertrophic growth in vitro. Supporting data from mice subjected to transverse aortic constriction demonstrated protection against cardiac hypertrophy upon PDE2 inhibition in vivo with BAY60-7550. The antihypertrophic effects recognized after PDE2 inhibition were attributed to cAMP-induced activation of PKA type II subset. The latter subsequently increases NFAT phosphorylation, thereby preventing its nuclear translocation and thus prohibiting hypertrophic signalling [18]. In a different setting, Baliga et al., also reported hindered adverse cardiac remodeling responses upon PDE2 blockade with BAY60-7550. In an alternative manner, PDE2 inhibition promotes cGMP signalling and ameliorates pathological left ventricular hypertrophy and fibrosis, improving cardiac contractility in a pressure overload heart failure model or post sympathetic hyperactivation. Using multiple transgenic animal models, the authors concluded that PDE2 inhibition preferentially enhances NO/sGC/cGMP signalling rather than ANP or BNP/pGC/cGMP cascades in heart failure settings to mediate these protective effects [19]. Other mechanisms have also been proposed, highlighting a role PDE2 in regulating the PLCε/PI4P-mediated hypertrophic pathway. PDE2 inhibition has been hypothesized to promote PKA-dependent phosphorylation and thereby inhibition of PLCε. Diminished PLCε-induced PI4P hydrolysis in the Golgi apparatus thus abrogates hypertrophic signalling [179].

In summary, recent studies seem to support that PDE2 inhibition promotes antihypertrophic responses. Nevertheless, the conflicting findings reviewed here, indicate that the role of PDE2 in hypertrophic remodeling should be conclusively verified and careful experimental designs should be constructed. Of course, disparate experimental methods and conditions may underlie the observed incoherent results [180], yet PDE2 is evidently a valuable therapeutic target to preclude cardiac remodeling events.

### 4.3. Myocardial Infarction (MI)/Reperfusion Injury

In patients suffering from MI, ischemia duration is critical, influencing the extent of myocardial hypoxia and cell necrosis. Management of myocardial infarcts remains a challenge in the cardiovascular field as with current interventions reperfusion injuries are difficult to circumvent. Post reperfusion, sarcolemmal rupture often occurs during the first few minutes upon restoring blood flow, thereby activating apoptotic and necrotic processes in cardiomyocytes [181]. Also, mitochondrial dysfunction, due to excessive mitochondrial Ca^2+^ accumulation, mitochondrial permeability transition and increased ROS production, has been implicated to mediate necrotic processes in ischemia reperfusion injuries. Thereby, therapeutic strategies supporting mitochondrial function may ameliorate the reperfusion damage [182,183]. Rinaldi L. et al., showed that PDE2 inhibition could protect against cell necrosis during ischemia reperfusion. The group initially demonstrated that diminished mitochondrial cAMP content is detrimental and exacerbates reperfusion damage post ischemia. On the other hand, overexpression of sAC improves cardiac cell viability [184]. Elevated mitochondrial cAMP content subsequently promotes PKA-dependent phosphorylation and activation of cytochrome c oxidase, enhancing ATP synthesis and attenuates ROS production as reported previously [184,185]. PDE2 inhibition during reperfusion and ischemia resulted in a specific increase in mitochondrial cAMP content, improved cell survival and accelerated recovery of cytosolic Ca^2+^ homeostasis [184].

Another study investigated if endogenously elevated ANP levels could limit infarct size after subjecting KO mice with endothelial GC-A deletion to MI [186]. NP-mediated GC-A/cGMP signalling was previously demonstrated to improve endothelial barrier properties downstream of cGMP-dependent kinase 1 (cGK1) under acute inflammatory conditions [187]. Unexpectedly, myocardial infarct size and immune cell infiltration two days after MI were attenuated in KO animals. These intriguing findings were attributed to PDE2 upregulation in the left ventricular myocardium, induced by hypoxia-mediated secretion of the inflammatory cytokine TNF-α. Elevated ANP levels in the diseased myocardium of WT animals promoted PDE2-cGMP/cAMP crosstalk, thus diminishing endothelial cAMP content. Reduced cAMP levels thereby hindered proper cardiac endothelial barrier function and induced capillary hyperpermeability. Endothelial GC-A deletion protected from PDE2 crosstalk mechanisms, attenuating infarct size. The authors therefore concluded that endothelial PDE2 inhibition might provide beneficial effects after MI [186].

### 4.4. Angiogenesis

Angiogenesis is a critical process coordinating new vessel formation from preexisting ones to maintain a sufficient oxygen supply during cardiac developmental and remodeling processes. Although the heart size remains rather stable during adulthood, the heart still retains a capacity for growth to sustain the cardiac output following physiological and pathophysiological increases in workload. In physiological reversible hypertrophy, arising during pregnancy or exercise training, proportional increases in cardiomyocyte size and angiogenic vessel development is achieved to preserve the oxygen supply. On the other hand, impaired angiogenic vessel enlargement and sprouting is a typical feature in pathological cardiac remodeling [188,189,190]. Angiogenesis inhibition in animal models of overload-induced cardiac hypertrophy resulted in early onset of heart failure [190]. To the contrary, delayed heart failure development was recognized after promoting angiogenic mechanisms [191].

The cardiomyocyte signalosome includes multiple cardiokines such as the vascular endothelial growth factor (VEGF), which promotes angiogenic processes via induction of endothelial cell proliferation and migration. Elevated cAMP levels in endothelial cells have been shown to inhibit VEGF-induced proliferation. A role for PDE2 and PDE4 was established to regulate this process in noncardiac endothelial derived cells [172]. So far, only the use PDE5 inhibitors, e.g., vardenafil, has been evaluated in a unilateral hindlimb ischemia model which showed that PDE5 inhibition enhances ischemia-induced angiogenesis [192]. Therefore, it would be interesting to study the role of PDEs, including PDE2 in ischemic cardiac diseases settings.

In vitro investigations in human umbilical vein cells (HUVEC) illustrated that PDE2 inhibition resulted in elevated cAMP levels after stimulation with VEGF. In turn, cell proliferation, entry to G2/M cell cycle phase and also cell migration were all hindered. Additionally, simultaneous inhibition of PDE2 and PDE4 in chicken embryos reduced angiogenesis in vivo [172]. Another study conducted by Diebold et al., also highlighted a role of PDE2 in ROS-induced endothelial cell proliferation and migration. PDE2 overexpression in HUVEC increased the activation of the GTPase Rac1, triggering NADPH oxidases to enhance ROS production. The latter consequently promoted cell proliferation and in vitro capillary formation. On the other hand, PDE2 inhibition or downregulation prevented ROS-induced HUVEC proliferation in vitro, vessel sprouting in mouse aortic explants ex vivo and angiogenesis in subcutaneous HUVEC Matrigel plug implantations in vivo [193]. Collectively, these studies could demonstrate a prominent role for PDE2 to modulate vascular dynamics. Nevertheless, further investigations are required to evaluate PDE2 as a therapeutic target in cardiovascular hypoxia and ischemic conditions stimulating angiogenesis.

### 4.5. Inflammation/Sepsis

After acute MI, hypoxia and elevated cytokine levels induce leukocytes infiltration in the myocardial tissue prompting cardiac inflammation. Neutrophils initiate the acute inflammatory response, whereas excessive infiltration increases the release of inflammatory mediators and kinases, contributing to tissue injury [186]. In HUVEC, TNF-α was shown to induce an increase in PDE2 expression levels. Likewise, in postcapillary venules within the mouse cremaster muscle, TNFα also promoted PDE2 expression and enhanced PDE2-mediated cGMP/cAMP crosstalk upon NP treatment. Augmented cAMP hydrolysis thereby provoked hyperpermeability and induced endothelial barrier dysfunction promoting neutrophil infiltration after MI. To the contrary, genetic GC-A deletion and pharmacological PDE2 inhibition prevented ANP-induced hyperpermeability in the TNF-α treated mice. In conclusion, PDE2 inhibition provides protective anti-inflammatory effects after MI limiting endothelial barrier permeability and leukocyte infiltration [186].

In line with these results, Seybold et al., also demonstrated TNF-α-induced increase in PDE2 expression in HUVECs under septic conditions [153]. Sepsis is a systemic inflammatory response characterized by increased endothelial barrier permeability due to enhanced TNF-α and thrombin secretion. TNF-α and thrombin promote platelet and leukocyte adhesion and provoke the secretion of inflammatory cytokines. The study demonstrated that TNF-α stimulated PDE2 expression upon activation of p38 mitogen-activated protein kinase (MAPK). PDE2 upregulation contributed to destabilize the endothelial barrier integrity and sensitized HUVECs towards the permeability-increasing agent thrombin. On the other hand, PDE2 selective inhibition significantly reduced edema formation in mice lungs and reduced lung permeability. At the molecular level, PDE2 inhibition also reduced TNF-α and thrombin-mediated alterations in endothelial F-actins and redistribution of VE-cadherin [153].

Furthermore, Neviere et al., indicated that PDE2 inhibition in septic cardiac fibers was beneficial as it limited sepsis-induced myocardial dysfunction and improved mitochondrial respiration [194]. Since sAC-induced cytochrome c oxidase phosphorylation is PKA-dependent, PDE2 inhibition partially increased cytochrome c oxidase subunit IV-1 protein phosphorylation in septic cardiac fibers. This led to an increase in respiratory control ratios, improved myocardial oxygen uptake efficiency and mitochondrial respiration. Moreover, perfusion of isolated septic hearts with the PDE2 inhibitor BAY60-7550 increased left ventricular developed force. Consistently, pretreatment with BAY60-7550, prior to a cecal ligation puncture procedure to induce sepsis, also significantly reduced coronary perfusion pressure and improved cardiac efficiency in septic mice in vivo [194]. Collectively, PDE2 inhibition improves myocardial function in septic hearts by maintaining endothelial barrier integrity and improving mitochondrial respiration.

### 4.6. Pulmonary Hypertension

Pulmonary hypertension (PH) affects the pulmonary vasculature due to the sustained elevation of pulmonary arterial pressure and remodeling of small pulmonary arteries [195,196]. PH is often associated with right ventricular heart failure and patients frequently exhibit sever signs of dyspnea [197]. Thereby, according to the National Heart Lung and Blood Institute of the National Institutes of Health (NIH), heart failure is one of the most common causes of death in patients with PH.

The most effective strategy to alleviate PH symptoms is to promote both cGMP and cAMP signalling enhancing vasodilation [196,198,199]. While PDE5 inhibitors have been extensively studied for clinical use to treat PH [198,200,201], PDE2 has not been comprehensively investigated and only one study demonstrated that PDE2 inhibition may ameliorate PH [196]. In this study, the authors demonstrated that PDE2 inhibition with BAY60-7550 prevented the onset of both hypoxia- and bleomycin-induced PH. BAY60-7550 treatment reduced the right ventricular systolic pressure, right ventricular hypertrophy and limited the number of muscularized pulmonary arteries. Protective effects achieved upon PDE2 inhibition were further augmented in the presence of cGMP- (sildenafil, inorganic nitrate) or cAMP- (i.e., treprostinil) enhancing agents. PDE2 inhibitor BAY60-7550 did not show any effect on mice lacking the NP GC-A receptor (NPR-A), but it showed positive effects on mice treated with L-NAME (NOS inhibitor). Thus, the positive effect of PDE2 inhibition alone was dependent on intact NP bioactivity, but not on NO-dependent signalling. Accordingly, PDE2 inhibition was demonstrated to show pronounced effects on NP-driven cGMP signalling in PH. In contrast to heart failure with upregulated PDE2 expression [16], PDE2 mRNA and protein expression are reduced in pulmonary artery smooth muscle cells from pulmonary arterial hypertension patients and pulmonary arteries from rats with hypoxia-induced PH [196]. Nevertheless, although this study provides evidence for the therapeutic benefits of PDE2 inhibition in PH, both in vitro and in vivo, the role of PDE2 in the pulmonary function is to be further assessed in order to acquire a deeper insight regarding PDE2-mediated effects in PH-related pathologies.

## 5. Clinical Perspectives

Despite the evident compelling role of PDE2 in various cardiac and vascular disease settings, to our knowledge no clinical trials regarding the use of PDE2 inhibitors in CVDs have been performed. Nevertheless, it is of interest to review ongoing clinical use of PDE2 inhibitors in other diseases. Considering the abundant expression of PDE2 in the brain, the use of selective PDE2 inhibitors has been extensively investigated to promote anxiolytic and antidepressant-like effects [15,202]. Additionally, other studies focused to address cognition enhancing effects of PDE2 inhibitors in aged and Alzheimer’s disease animal models [203,204]. Despite the promising data in preclinical settings, the use of PDE2 inhibitors is currently not clinically approved. The selective PDE2 inhibitor PF-05180999 was originally developed by Pfizer for treatment of cognitive deficits associated with schizophrenia. Thereafter, PF-05180999 was tested in Phase I clinical trials for the treatment of migraines. However, the trials were terminated prematurely due to safety concerns [15,205]. Another selective PDE2 inhibitor, TAK-915, was inspected in Phase I trials to determine appropriate dose selection for cognitive impairment therapies in schizophrenia [15,206,207]. Beyond the brain, PDE2 was shown to be upregulated in cancerous cells. Therefore, the PDE2A/PDE5A inhibitor exisulind has been evaluated in several clinical trials as it promotes precancerous/cancerous cells apoptosis. Nevertheless, the FDA also disapproved the use of exisulind due to deficiencies in safety and efficacy [15,208]. Thereby, the development of safe and potent PDE2 inhibitors remains challenging at the current stage and is to be further investigated in the future.

## 6. Conclusions

In conclusion, studies in CVD models revealed both beneficial and detrimental effects of PDE2 activity. The controversial PDE2 effects seem not only to be dependent on the activity of PDE2 in distinct cardiac cell types, but also in different subcellular compartments. While the enhanced PDE2 activity in one specific subcellular compartment seems to be beneficial, detrimental responses in other microdomains may be disposed. This highlights the degree of complexity but yet indispensable function of cardiac PDE2 compartmentalization. Maintenance of physiological PDE distribution is thereby elemental for the proper function of the myocardium.

Nevertheless, currently both, the activation and inhibition of PDE2, provide solid beneficial outcomes in cardiac pathological disease settings. Further investigations should aim to provide conclusive pharmacological effects upon modulating PDE2 activity in vivo. In addition, a more comprehensive understanding of the role of distinct PDE2 splice variants is required. Development of specific PDE2 agonists and generation of cell type-restricted PDE2 deficient animal models could further elucidate physiological as well as pathophysiological roles of PDE2. Implementing the use of such novel tools will therefore provide evidence to whether PDE2 activity is beneficial or detrimental in cardiovascular diseases.

## Abbreviations

AC	Adenylyl cyclase
AhR	Aryl hydrocarbon receptor
ANP	Atrial natriuretic peptide
AKAP	A-kinase anchoring protein
β-AR	β-adrenoceptor
BNP	Brain natriuretic peptide
cAMP	3′,5′-cyclic adenosine monophosphate
cGMP	3′,5′-cyclic guanosine monophosphate
CNGCs	Cyclic nucleotide gated ion channels
CNP	C-type natriuretic peptide
CVD	Cardiovascular disease
DRP1	Dynamin related protein 1
ECC	Excitation-contraction coupling
EPAC	Exchange proteins directly activated by cAMP
GPCR	G protein coupled receptors
HUVEC	Human umbilical vein cells
LTCC	L-type Ca^2+^ channel
MI	Myocardial infarction
MPT	Mitochondrial permeability transition
MYBPC	Myosin-binding protein C
NP	Natriuretic peptide
NOS	Nitric oxide synthase
PDE	Phosphodiesterase
pGC	Particulate guanylyl cyclases
PH	Pulmonary hypertension
PLB	Phospholamban
POPDC	Popeye-domain-containing proteins
RyR	Ryanodine receptors
sAC	Soluble adenylyl cyclases
sGC	Soluble guanylyl cyclases
SNP	Sodium nitroprusside
SR	Sarcoplasmic reticulum
TNF-α	Tumor necrosis factor-α
VEGF	Vascular endothelial growth factor
VSMC	Vascular smooth muscle cell
VEC	Vascular endothelial cell
